# Characterizing arginine, ornithine, and putrescine pathways in enteric pathobionts

**DOI:** 10.1002/mbo3.1408

**Published:** 2024-04-01

**Authors:** Ian M. Lillie, Charles E. Booth, Adelaide E. Horvath, Matthew Mondragon, Melinda A. Engevik, Thomas D. Horvath

**Affiliations:** ^1^ Department of Materials Science & Engineering Cornell University Ithaca New York USA; ^2^ Department of Pathology & Immunology Baylor College of Medicine Houston Texas USA; ^3^ Department of Pathology Texas Children's Hospital Houston Texas USA; ^4^ Department of Regenerative Medicine & Cell Biology Medical University of South Carolina Charleston South Carolina USA; ^5^ Department of Biology & Biochemistry University of Houston Houston Texas USA; ^6^ Department of Mathematics University of Houston Houston Texas USA; ^7^ Department of Microbiology & Immunology Medical University of South Carolina Charleston South Carolina USA

**Keywords:** bacteria, genome, LC‐MS/MS, targeted metabolomics

## Abstract

Arginine‐ornithine metabolism plays a crucial role in bacterial homeostasis, as evidenced by numerous studies. However, the utilization of arginine and the downstream products of its metabolism remain undefined in various gut bacteria. To bridge this knowledge gap, we employed genomic screening to pinpoint relevant metabolic targets. We also devised a targeted liquid chromatography‐tandem mass spectrometry (LC‐MS/MS) metabolomics method to measure the levels of arginine, its upstream precursors, and downstream products in cell‐free conditioned media from enteric pathobionts, including *Escherichia coli*, *Klebsiella aerogenes*, *K. pneumoniae*, *Pseudomonas fluorescens*, *Acinetobacter baumannii*, *Streptococcus agalactiae*, *Staphylococcus epidermidis*, *S. aureus*, and *Enterococcus faecalis*. Our findings revealed that all selected bacterial strains consumed glutamine, glutamate, and arginine, and produced citrulline, ornithine, and GABA in our chemically defined medium. Additionally, *E. coli*, *K. pneumoniae*, *K. aerogenes*, and *P. fluorescens* were found to convert arginine to agmatine and produce putrescine. Interestingly, arginine supplementation promoted biofilm formation in *K. pneumoniae*, while ornithine supplementation enhanced biofilm formation in *S. epidermidis*. These findings offer a comprehensive insight into arginine‐ornithine metabolism in enteric pathobionts.

## Graphical abstract

The amino acid L‐arginine is converted by bacteria into a variety of downstream metabolites, including citrulline, agmatine, ornithine, putrescine, and gamma‐aminobutyric acid (GABA). Our study shows that both Gram‐negative bacteria (*Escherichia coli, Klebsiella pneumoniae, Klebsiella aerogenes, Pseudomonas fluorescens*, and *Acinetobacter baumannii*) and Gram‐positive bacteria (*Streptococcus agalactiae, Staphylococcus epidermidis, Staphylococcus aureus*, and *Enterococcus faecalis*) consume arginine, glutamine, and glutamate, and generate citrulline, ornithine, and GABA. This suggests that these pathways are conserved and essential within gut bacteria. We also found that *Escherichia coli, Klebsiella pneumoniae, Klebsiella aerogenes*, and *Pseudomonas fluorescens* generate the arginine intermediate agmatine and produce the polyamine putrescine. These findings indicate that arginine is a crucial amino acid for gut bacteria.

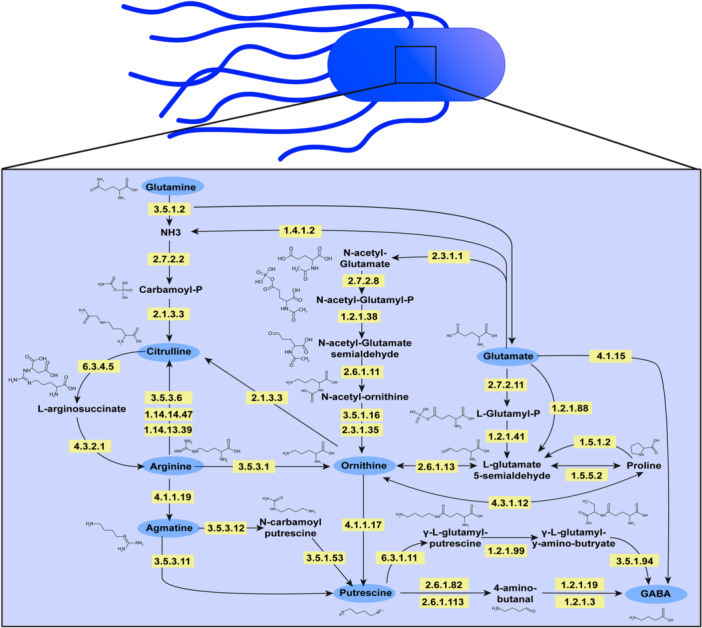

## 1 INTRODUCTION

**Figure 1:**
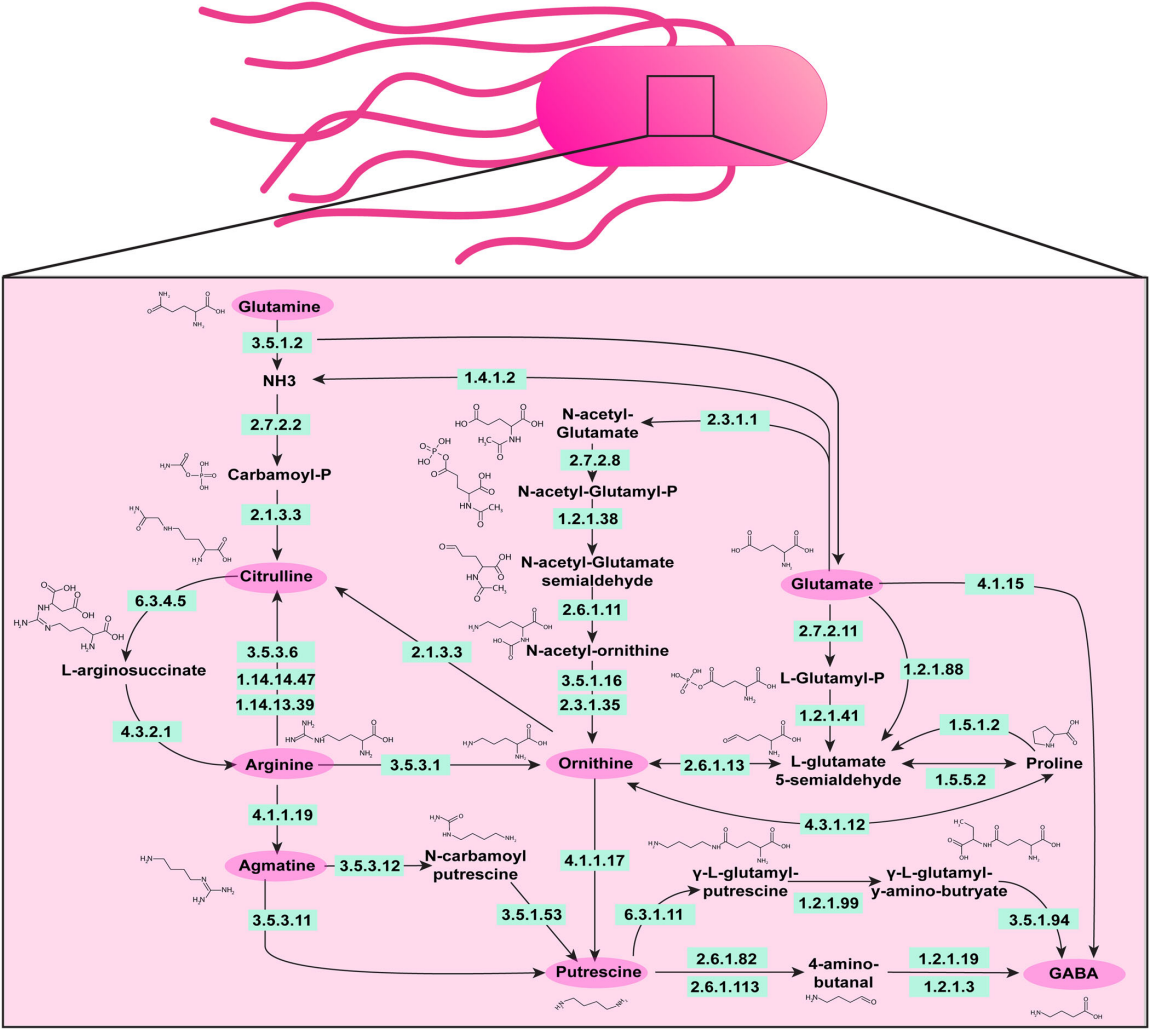
Diagram of the known pathways involved in arginine metabolism in bacteria. Adapted from KEGG Pathway Database (https://www.genome.jp/kegg/pathway.html).

Arginine is an important and versatile amino acid that can be utilized as both a carbon and nitrogen source for bacteria (Dhodary et al., 2022; Wu & Morris, 1998). In the gut, L‐arginine can be generated from the breakdown of proteins found in meat, fish, dairy products, and nuts (Hu et al., 1998; Singh et al., 2019; Visek, 1986). L‐arginine can then be transported by cationic amino acid transporters (CAT) into bacteria and be converted to agmatine, ornithine, citrulline, and polyamines. Arginine transporters and arginine metabolic pathways have been identified in multiple gut bacteria and have been well studied in the model organisms *Escherichia coli* and *Bacillus subtilis* (Charlier & Bervoets, 2019; Ginesy et al., 2015; Xiong et al., 2016; Yang et al., 2021). Arginine is a potential energy source for bacteria as the breakdown of arginine to ornithine and ammonium is coupled to the generation of metabolic energy in the form of ATP (Pols et al., 2021). Arginine can also be converted into the polyamine putrescine and subsequently into the neurotransmitter gamma‐aminobutyric acid (GABA) (Figure 1). Due to its importance in the cell, arginine can be *de novo* synthesized by bacteria from several compounds, such as the amino acids glutamate and glutamine. In the enteric pathogens Enterohemorrhagic *Escherichia coli* (EHEC) and *Citrobacter rodentium*, arginine has been shown to up‐regulate virulence genes (Menezes‐Garcia et al., 2020) and arginine may regulate many aspects of bacteria physiology. Despite the global importance of arginine in bacteria, arginine utilization and the downstream products of arginine metabolism are not fully defined in several gut bacteria.

Using selected‐reaction monitoring (SRM)‐based targeted metabolomics approach is a potent strategy to quantify the absolute concentrations of bacterial‐derived metabolites in cultures because of the technique's high degree of sensitivity, selectivity and specificity, and batch‐to‐batch reproducibility. The quantitative bioanalytical method described here is based on the use of external calibrators prepared across a linear dynamic concentration range of ~1000‐fold (0.977–1000 ng/ml), and a consistent deuterated internal standard (IS) concentration (225 ng/ml) being added to all calibrator, blank controls, and biological specimens. The absolute concentration of the metabolite content of each sample was performed using a linear regression model with an applied 1/x weighting factor.

In this study, we focused on enteric pathobionts; bacteria that can be found in the gut microbiota under normal conditions but can act as pathogens and induce inflammation or septicemia (i.e., bloodstream infections) when conditions are favorable for the pathobiont. We used genomic screening to identify relevant metabolic targets and developed a targeted liquid chromatography‐tandem mass spectrometry (LC‐MS/MS)‐based metabolomics method to quantify arginine and its upstream precursors and downstream products in cell‐free conditioned bacterial media supernatant samples. This combination of microbial genomics screening with a targeted metabolomics method offers an in‐depth analysis of arginine metabolism in enteric pathobionts.

## 2 METHODS

### 2.1 Genome analysis and bacterial culturing

To assess the distribution of arginine pathways among bacterial species, we queried the Integrated Microbial Genomes (IMG) database v5.0 (http://img.jgi.doe.gov) (accessed July 7, 2023) (Chen et al., 2021), which is available through the Joint Genomes Institute (JGI) (Version 6.0) (Chen et al., 2021). Enzyme Commission numbers (EC) for bacterial pathways were examined in all annotated genomes (Table 1). ECs were input into the gene search tool of the IMG database and the bacterial genomes were binned for analysis. High‐quality drafts (>90% complete with the presence of the 23 S, 16 S, and 5 S rRNA genes, as well as at least 18 tRNAs, and with less than 5% contamination) and finished (single contiguous sequences without gaps and less than one error per 100,000 base pairs) were included for analysis. In the IMG database, we identified 5315 genomes of *S. aureus*, 4201 genomes of *E. coli*, 1306 genomes of *A. baumannii*, 1235 of *K. pneumoniae*, 725 genomes of *S. agalactiae*, 421 genomes of *E. faecalis*, 180 genomes of *S. epidermidis*, 145 genomes of *P. fluorescens*, and 112 genomes of *K. aerogenes*. Duplicate genomes were removed from the analysis. Genomes that harbored at least one gene copy of a specified EC were considered to possess that function. To determine how many bacteria from a given species possessed an EC function, we used the following equation:

(1) $$\mathrm{Bacteral}\;\mathrm{genomes}\;\mathrm{harboring}\;\mathrm{an}\;\mathrm{EC}\;\left(\%\right)=\frac{\left({\mathrm{Bacterial}\;\mathrm{genomes}}_{+\mathrm{EC}}\right)}{\left({\mathrm{Bacterial}\;\mathrm{genomes}}_{\mathrm{total}}\right)}*100$$

**Table 1:** Optical density at 600 nm (OD_600nm_) for bacteria grown in ZMB1 for 20 h.

Bacteria	Strain	OD_600nm_	OD_600nm_	OD_600nm_
*Escherichia coli*	NCTC 13846	4.36	3.83	4.27
*Klebsiella pneumoniae*	ATCC 9101	8.79	8.22	8.65
*Klebsiella aerogenes*	ATCC 13048	6.89	6.36	6.36
*Pseudomonas fluorescens*	CB1	6.37	6.79	6.89
*Acinetobacter baumannii*	ATCC 19606	4.57	4.58	4.55
*Streptococcus agalactiae*	NCIMB 701348	5.69	5.12	5.13
*Staphylococcus epidermidis*	ATCC 51625	5.57	6.06	6.04
*Staphylococcus aureus*	NCTC 12493	5.51	5.44	5.48
*Enterococcus faecalis*	ATCC 29212	5.75	5.70	5.71

For in vitro assessment of the arginine pathway, we selected commercially available strains capable of being grown in a chemically defined bacterial medium. The following commercially available pathobionts were used in this study: *Klebsiella aerogenes* ATCC 13048, *K. pneumoniae* ATCC 9101, *Escherichia coli* ATCC BAA‐2452, *Staphylococcus epidermidis* ATCC 51025, *S. aureus* NCTC 12493, *Pseudomonas fluorescens* CB1, *Acinetobacter baumannii* ATCC 19606, *Streptococcus agalactiae* ATCC 13813, and *Enterococcus faecalis* ATCC 29212.

All bacteria were grown aerobically overnight at 37°C in brain‐heart‐infusion (BHI) broth. After confirming growth, cultures were centrifuged at 6000 x *g* for 5 min to pellet bacteria. In each instance, bacterial pellets were washed 3x with sterile PBS to remove traces of the rich media. After the final wash, the bacterial pellet was resuspended in an equal volume of a chemically defined culture medium called ZMB1 (Engevik et al., 2023; Horvath et al., 2023a; Zhang et al., 2009) and subcultured to an optical density (OD_600nm_) of 0.1 in 5 ml of ZMB1. All cultures were grown in biological triplicate aerobically at 37°C. After 20 h of incubation, the bacterial growth was assessed by measuring OD_600nm_. After measuring the OD_600nm_, cultures were centrifuged at 6000 x *g* for 5 min to pellet the bacteria, and the conditioned media supernatant samples were sterile filtered using 0.2 μm filters and processed for targeted metabolomics‐based bioanalysis.

For biofilm analysis, all bacteria were grown in BHI and subcultured at an OD_600nm_ = 0.1 in Tryptic Soy Broth (TSB) with a range (1 mM–1 nM) of Arginine (FisherSci # A15738‐14) or Ornithine (FisherSci# A12111‐14). Bacteria were grown in 96‐well plates for 72 h and biofilm formation was assessed by crystal violet staining as previously described (Engevik et al., 2021a).

## 3 SOLVENTS, CHEMICALS AND DURABLE SUPPLIES

Optima™ LC/MS‐grade water, acetonitrile (ACN), and formic acid (FA) were obtained from Fisher Scientific (Waltham, MA, USA). MS‐grade ammonium formate was obtained from Millipore‐Sigma (Burlington, MA, USA). Authentic analytical reference standards for arginine, agmatine, ornithine, N‐carbamoylputrescine, putrescine, and citrulline were all purchased from Millipore‐Sigma. Deuterated internal standard (IS) compounds, including d7‐arginine, d7‐ornithine, d4‐putrescine, and d7‐citrulline were all purchased from CDN Isotopes (Pointe‐Claire, Quebec, Canada). Chromatographic separations were performed using a Supelco Ascentis® Express HILIC (150 mm x 2.1 mm, 2.7 µm, 90 Å pore) analytical column from Millipore‐Sigma.

## 4 LC‐MS/MS EQUIPMENT

The LC‐MS/MS system was comprised of a Shimadzu Nexera X2 MP Ultrahigh‐Performance Liquid Chromatography (UHPLC) system (Kyoto, Japan) coupled to a SCIEX QTRAP 6500 hybrid triple‐quadrupole/linear ion trap MS system (Framingham, MA, USA). Infusions of SCIEX PPG‐based positive and negative mode instrument calibration standards were used to perform routine instrument calibrations according to the manufacturer's specifications for sensitivity, mass error, and resolution (*m/z* 0.6–0.8 at full width at half max (FWHM) for unit/unit quadrupole resolution) in each polarity. For this method, the QTRAP 6500 was operated in the low mass mode, and instrument calibrations were performed using PPG ions that span *m/z* 59.05–*m/z* 1196.883 in positive ionization mode, and *m/z* 44.998–*m/z* 1223.845 in negative ionization mode (See Page 163 of the SCIEX System User's Guild (RUO‐IDV‐05‐2095‐A; Aug 2015) for more details). Operational control of the LC‐MS/MS was performed with Analyst (Ver. 1.6.2), and quantitative analysis was performed using MultiQuant (Ver. 3.0.6256.0).

## 5 LC‐MS/MS METHOD FOR ARGININE POLYAMINE METABOLITES

Individual Internal Standard (IS) stock solutions for d7‐arginine, d7‐ornithine, d4‐putrescine, and d7‐citrulline were each prepared at concentrations of 5.0 mg/ml in water. Individual stock solutions of the analytes arginine, agmatine, ornithine, N‐carbamoylputrescine, putrescine, and citrulline were each prepared at concentrations of 10 mg/ml in water.

A 100 ml volume of a solvent solution consisting of 20% ethanol, 72% acetonitrile (ACN), and 8% water with 0.01% formic acid (FA) was prepared. Then, a 5 μl volume of each deuterated IS stock solution was added to the solvent solution to produce an IS Solution‐A (ISS‐A) at a concentration of 250 ng/ml for each deuterated IS compound. The ISS‐A was used in the final preparation of the bacterial culture samples. An IS Solution‐B (ISS‐B) was prepared at a concentration of 225 ng/ml of each deuterated standard by diluting a 4.5 ml volume of the ISS‐A solution with a 0.5 ml volume of the same solvent solution EtOH:ACN:H_2_O:FA (20:72:8:0.01, *v:v:v:v*). The ISS‐B was used as the diluent in the preparation of the combined intermediate solution and each of the calibration standards.

A combined intermediate was prepared by mixing a 10 μl volume of each of the arginine, agmatine, ornithine, N‐carbamoylputrescine, putrescine, and citrulline stock solutions into a 940 μl volume of ISS‐B. This intermediate solution was used to prepare the calibration standards through a fourfold serial dilution procedure to produce calibrators with targeted metabolite concentrations of 1000, 250, 62.5, 15.6, 3.90, and 0.977 ng/ml using ISS‐B as the diluent. A 10 μl volume of each calibrator was injected onto the LC‐MS/MS system in an ascending concentration sequence at the beginning of the sample queue to produce metabolite‐specific calibration curves that were used to determine the absolute concentrations of the targeted metabolites contained in the bacterial‐derived specimens. For a given metabolite, a plot of the instrument response ratio (IRR = Area_Analyte_/Area_IS_) for each calibrator is plotted on the y‐axis against the nominal concentration of the calibration standards on the x‐axis, then a linear regression analysis (with 1/x weighting) is performed to generate a line of best fit, that is, a calibration curve for that specific metabolite. The absolute concentration of each targeted metabolite in the biological specimens was computed using the IRR for a specific metabolite in a given sample, and the slope (m) and y‐intercept (b) from the calibration curve for that specific metabolite using the following equation:

(2) $$\left[\mathrm{metabolite}\right]\;\left(\mathrm{ng}/\mathrm{mL}\right)=\left(\frac{\mathrm{IRR}-b}{m}\right)$$

The quantitative analysis approach described directly above was automated for all calibrators, blanks, and blank controls (blank + IS samples, and unknown specimens) using the SCIEX Multiquant software package.

Cell‐free conditioned bacterial media samples were thawed on the benchtop at ambient room temperature and were then vortex‐mixed thoroughly. A 10 μl volume of each sample was diluted in a 90 μl volume of an ISS‐A solution, and each of the 10‐fold diluted samples was then briefly vortex‐mixed. Because arginine is present as a nutrient at a high concentration in the ZMBI growth medium, each of the 10‐fold diluted samples from above was diluted an additional 50‐fold by transferring a 5 μl sample volume into a second tube that contained a 245 μl volume of ISS‐A solution; the overall dilution factor for this second set of samples was 500‐fold. All samples were transferred to fresh autosampler vials, and a 10 μl sample volume was injected for each onto the LC‐MS/MS system for analysis.

Arginine pathway metabolite separations are based on hydrophilic interaction chromatography (HILIC) using mobile phase A (MPA) and mobile phase B (MPB) solutions consisting of ACN:water (95:5, *v:v*) with 10 mM ammonium formate and 2% formic acid (FA), and ACN:water (1:1, *v:v*) with 10 mM ammonium formate and 2% FA, respectively, and a needlewash solution consisting of ACN:water (1:1, *v:v*). Chromatographic separations were performed using an Ascentis® Express HILIC, 2.7 μm (150 mm x 2.1 mm) analytical column (Supelco, Bellefonte, PA, USA). The mobile phase flowrate was 300 μl/min, autosampler trays were chilled to 4°C, the column was heated to 30°C, and the gradient elution program used was 0–2.5 min, 20% MPB; 2.5–10.5 min, 20%–70% MPB; 10.5–13 min, 70% MPB; 13–14 min, 70%–20% MPB; 14–18 min, 20% MPB, with a gradient cycle time of 18 min per sample. A TurboIonSpray® electrospray ionization (ESI) probe was installed in the IonDrive™ Turbo V™ ionization source attached to the MS system, and the QTRAP 6500 MS was operated in positive ionization mode using a multiple‐reaction monitoring (MRM) scan mode with the following instrumental conditions: IonSpray voltage of +5500 volts (V); Curtain gas (Cur) of 25 psi; Temperature (Temp) of 400°C; Source Gas 1 (GS1) of 50 psi; Source Gas 2 (GS2) of 50 psi; Collisionally‐activated dissociation (CAD) gas set at high; and the Q1 and Q3 quadrupole resolution settings were set to Unit/Unit.

## 6 RESULTS

**Figure 2:**
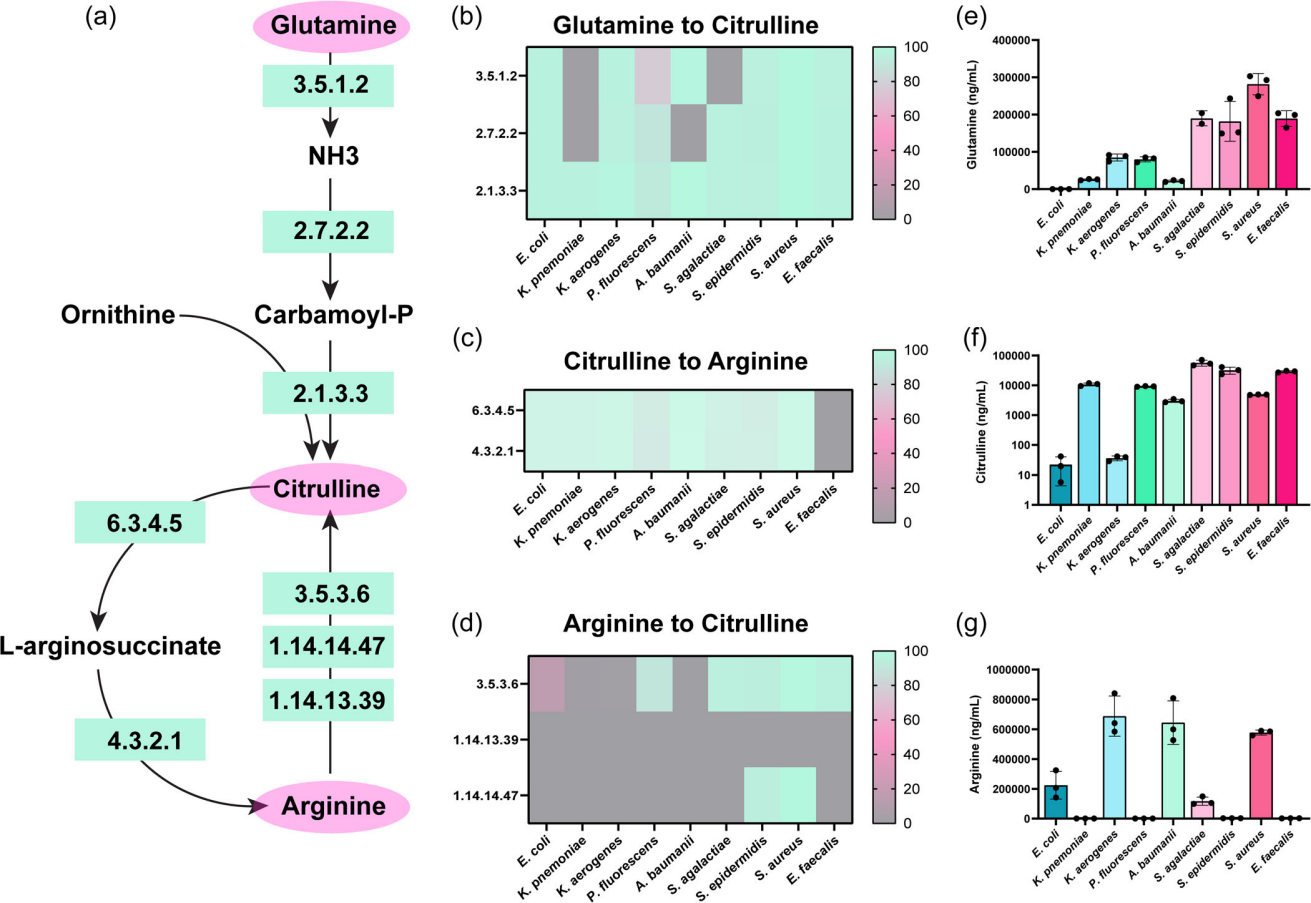
(a) Simplified diagram of the pathways to convert Glutamine to Arginine with the corresponding enzyme commission (EC) numbers responsible for these reactions. Percentage of bacterial genomes harboring ECs involved in the conversion of (b) Glutamine to Citrulline, (c) Citrulline to Arginine, (d) Arginine to Citrulline. LC‐MS/MS data of bacterial supernatant for the absolute concentrations of (e) Glutamine, (f) Citrulline, and (g) Arginine. *n* = 3; biological triplicate of bacteria samples. Each dot represents an individual data point.

To understand how pathobionts in the gut generate and use the amino acid arginine, we examined the known pathways of arginine metabolism in bacteria (Figure 1). We selected enteric pathobionts from the phyla γ‐Proteobacteria (*E. coli, K. pneumoniae, K. aerogenes, P. fluorescens, A. baumannii*), and Firmicutes *(S. agalactiae, S. epidermidis, S. aureus, E. faecalis*) and examined their genomes in the Integrated Microbial Genomes (IMG) database (http://img.jgi.doe.gov). Bacteria can generate arginine through several different pathways (Figure 1). One pathway is the conversion of glutamine to arginine. Glutamine can be converted to glutamate and ammonia (NH_3_) by EC 3.5.1.2 (glutaminase) then to citrulline via EC 2.7.2.2 (carbamate kinase) and EC 2.1.3.3 (ornithine carbamoyltransferase) (Figure 2a). We examined the enzymes required for this multi‐step conversion of glutamine to citrulline in the selected enteric bacterial genomes. We calculated the percentage of bacteria within a certain species that possessed a given EC based on the number of bacterial genomes that had the EC of interest, divided by the total number of bacterial genomes of that species (see Equation 1) (Figure 2b). We found that >80% of *E. coli, K. aerogenes, S. epidermidis, S. aureus* and *E. faecalis* genomes in the database encoded the enzymes necessary to convert glutamine to citrulline. The bacteria *K. pneumoniae, A. baumannii*, and *S. agalactiae* were found to lack one or more of these enzymes, suggesting that these bacteria cannot convert glutamine to citrulline through this specific pathway. However, some bacteria can use EC 2.1.3.3 to convert ornithine to citrulline and we found that all the bacterial species encoded this enzyme (Figure 2b).

Bacteria can convert citrulline into arginine via EC 6.3.4.5 (argininosuccinate synthase) and EC 4.3.2.1 (argininosuccinate lyase) (Figure 2a) and we found that all pathobionts except *E. faecalis* possessed these ECs (Figure 2c). Arginine can also be converted directly into citrulline through three enzymes (EC 3.5.3.6 [arginine deiminase], EC 1.14.14.47 [nitric oxide synthase oxygenase], or EC 1.4.13.39 [nitric‐oxide synthase]). We found *S. epidermidis* and *S. aureus* possessed 2 of these enzymes (Figure 2d); indicating that the majority of selected pathobionts were likely not converting arginine to citrulline. To identify the levels of glutamine, citrulline, and arginine experimentally, we grew representative gut pathobionts in a chemically defined medium (ZMB1) which supported the growth of all the bacteria (Table 1). We then collected cell‐free conditioned bacterial media to examine secreted compounds in our pathway and performed targeted LC‐MS/MS‐based metabolomics. The uninoculated medium at baseline contained 308 ± 9.1 μg/mL glutamine and 940 ± 16 μg/ml arginine). Targeted metabolomic‐based analysis revealed a net depletion of glutamine in all the bacterial cultures (Figure 2e). Of the microbes examined, the γ‐Proteobacteria members (*E. coli, K. pneumoniae K. aerogenes, P. fluorescens*, and *A. baumannii*) consumed the most glutamine. Consistent with decreased levels of culture glutamine, we found elevated levels of citrulline in all of the bacterial samples (Figure 2f), with Firmicutes members *S. agalactiae, S. epidermidis, S. aureus*, and *E. faecalis* generating the highest levels. Similar to the glutamine levels, we observed a general decrease in arginine compared to the uninoculated bacterial medium (Figure 2g); suggesting that all the bacteria consumed arginine from the medium.

**Figure 3:**
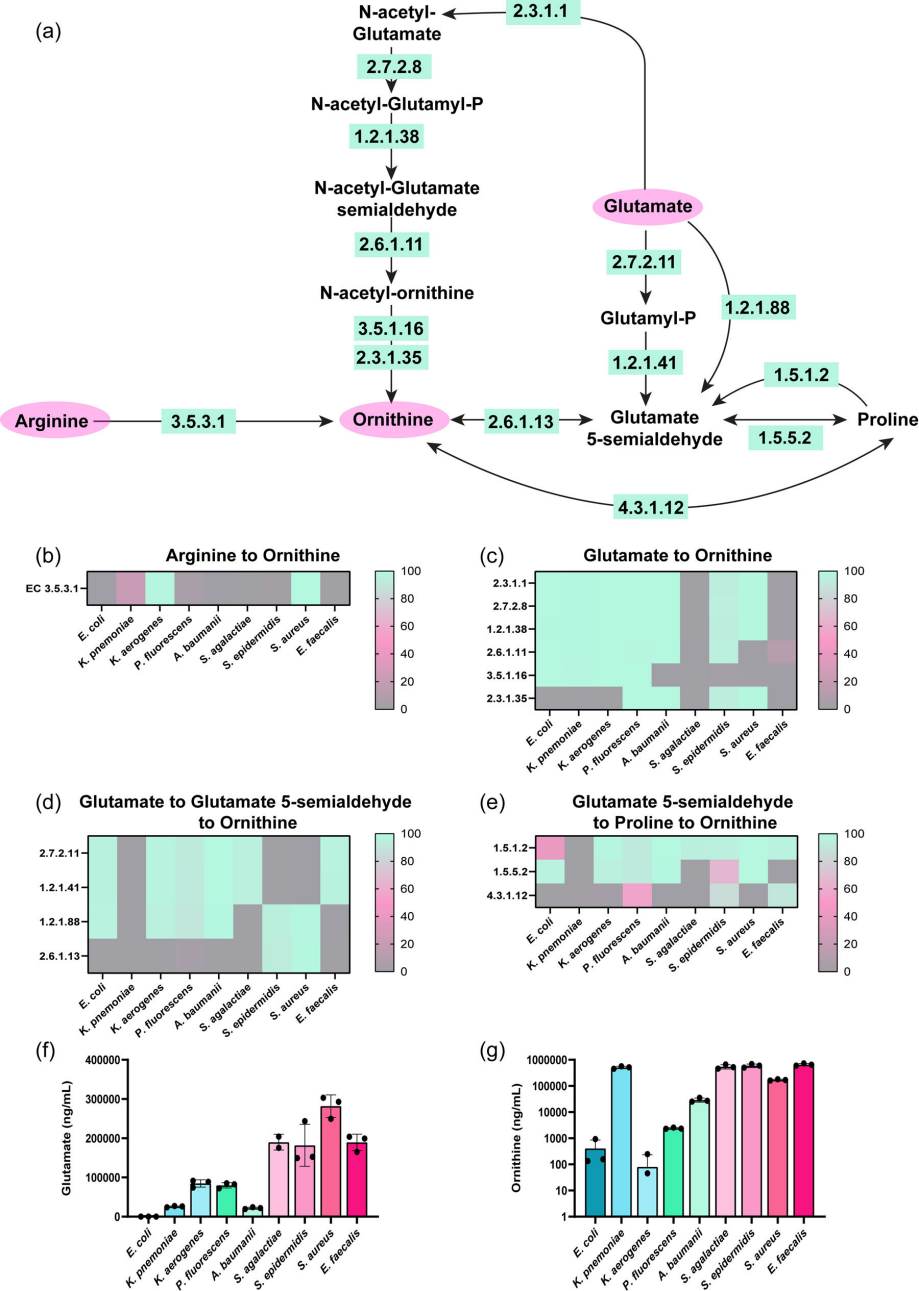
(a) Simplified diagram of the pathways to convert Arginine and Glutamate to Ornithine with the corresponding enzyme commission (EC) numbers responsible for these reactions. Percentage of bacterial genomes harboring ECs involved in the conversion of (b) Arginine to Ornithine, (c) Glutamate to Ornithine, (d) Glutamate to Glutamate‐5‐semialdehyde to Ornithine, (e) Glutmate‐5‐semialdhyde to Proline to Ornithine. Quantitative LC‐MS/MS‐based targeted metabolomics data of bacterial supernatant for the absolute concentrations of (f) Glutamate, and (g) Ornithine. *n* = 3; biological triplicate of bacteria samples. Each dot represents an individual data point.

Since we observed a general decrease in arginine, we next examined the downstream compounds of arginine metabolism. In bacteria, arginine can be converted to ornithine by EC 3.5.3.1 (arginases) (Figure 3a). Analysis of our enteric pathobiont genomes revealed that all of the *K. aerogenes* and *S. aureus* genomes harbored EC 3.5.3.1 (Figure 3b) and roughly one‐half of the *K. pneumoniae* genomes possessed this enzyme. These data indicate that only four of the selected bacteria were able to convert arginine to ornithine via a direct path. Ornithine can also be generated from glutamate via a five‐step reaction sequence involving EC 2.3.1.1 (glutamate N‐acetyltransferase), EC 2.7.28 (acetylglutamate kinase), EC 1.2.1.38 (N‐acetyl‐gamma‐glutamyl‐phosphate reductase), EC 2.6.1.11 (acetylornithine transaminase) and EC 3.5.1.16 (acetylornithine deacetylase)/EC 2.3.1.35 (glutamate N‐acetyltransferase)), or by a two to three‐step reaction via L‐ glutamate‐5‐semialdehyde (EC 2.7.2.11 (glutamate 5‐kinase), EC 1.2.1.41 (glutamate‐5‐semialdehyde dehydrogenase), EC 1.2.1.88 (l‐glutamate gamma‐semialdehyde dehydrogenase) and EC 2.6.1.13 (ornithine aminotransferase)) (Figure 3a). Genome analysis indicated that *E. coli, K. pneumoniae, K. aerogenes*, *P. fluorescens, A. baumannii*, and *S. epidermidis* possessed the ECs to fully convert glutamate to ornithine via the five‐step reaction sequence (Figure 3c).

Glutamate can also be converted to ornithine via the production of L‐glutamate‐5‐semialdehyde by either a two‐step reaction sequence (EC 2.7.2.11 (glutamate 5‐kinase) and EC 1.2.1.41 (glutamate‐5‐semialdehyde dehydrogenase)) or by a single step reaction (EC 1.2.1.88 (L‐glutamate gamma‐semialdehyde dehydrogenase)), followed by EC 2.6.1.13 (ornithine aminotransferase) (Figure 3a). Only *S. epidermidis* and *S. aureus* could convert L‐glutamate‐5‐semialdehyde to ornithine through this pathway (Figure 3d). All the pathobionts, except *K. pneumoniae* had the enzymes to convert glutamate to L‐glutamate‐5‐semialdehyde to proline (Figure 3e) and S. *epidermidis* and *E. faecalis* could convert L‐glutamate‐5‐semialdehyde to proline to ornithine (Figure 3e).

Based on these analyses, we would predict that our selected pathobionts would deplete glutamate and generate ornithine. To confirm our genome analysis, we used targeted LC‐MS/MS‐based metabolomics to examine the concentrations of glutamate and ornithine in our bacterial supernatants. The uninoculated bacteria medium had 274 ± 21 μg/ml of glutamate. After incubation with our bacteria, we found a significant reduction of glutamate in all the samples, with the most depletion observed in *E. coli* and *K. aerogenes* cultures (Figure 3f). Since all bacteria were predicted to generate ornithine, albeit through different pathways, we next examined the levels of ornithine in the cell‐free supernatants measured by our LC‐MS/MS method. We found that all of the bacteria generated high levels of ornithine in the cell‐free conditioned media relative to the inoculated ZMB1 medium (Figure 3g). Of the bacteria examined, *K. pneumoniae, S. agalactiae, S. epidermidis, S. aureus*, and *E. faecalis* secreted the highest concentrations of ornithine. These data suggest that ornithine is an important intermediate for these enteric pathobionts.

**Figure 4 (a):**
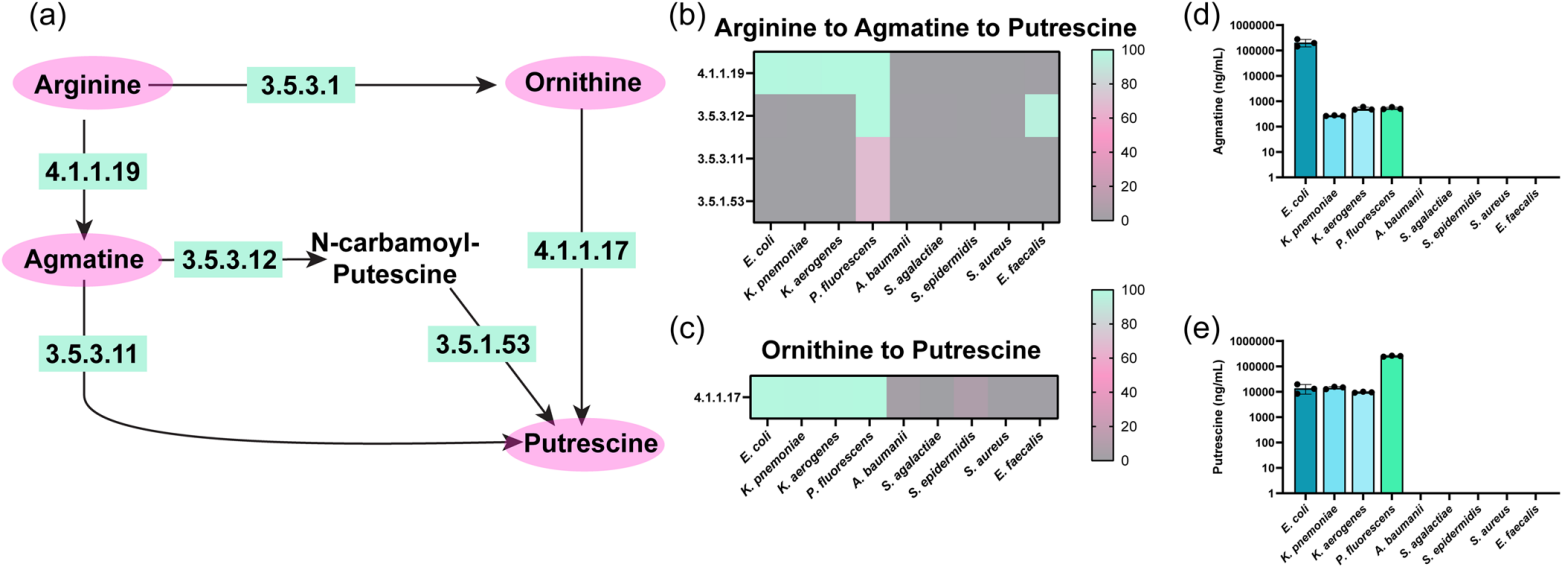
Simplified diagram of the pathways to convert Arginine to Agmatine and Ornithine and Agmatine to Putrescine with the corresponding enzyme commission (EC) numbers responsible for these reactions. Percentage of bacterial genomes harboring ECs involved in the conversion of (b) Arginine to Agmatine to Putrescine and (c) Ornithine to Putrescine. Quantitative LC‐MS/MS‐based targeted metabolomics data of bacterial supernatant for the absolute concentrations of (d) Agmatine, and (e) Putrescine. *n* = 3; biological triplicate of bacteria samples. Each dot represents an individual data point.

Arginine can also be converted to agmatine via EC 4.1.1.19 (arginine decarboxylase) and subsequently converted into putrescine by a one‐step reaction (EC 3.5.3.11 [agmatinase]), or a two‐step reaction sequence (EC 3.5.2.12 [6‐aminohexanoate‐cyclic‐dimer hydrolase] and EC 3.5.1.53 [N‐carbamoylputrescine amidase])(Figure 4a). Among our bacteria of interest, *E. coli, K. pneumoniae, K. aerogenes*, and *P. fluorescens* possessed EC 4.1.1.19 to generate agmatine (Figure 4b). However, only *P. fluorescens* genomes could convert agmatine to putrescine via the direct route (EC 3.5.3.11) and the two‐step reaction sequence (EC 3.5.3.12 and EC 3.5.1.53) routes (Figure 4b). Putrescine can also be generated from ornithine via EC 4.1.1.17 (ornithine decarboxylase) (Figure 4c). We found that *E. coli, K. pneumoniae, K. aerogenes*, and *P. fluorescens* all had the enzyme to convert ornithine to putrescine. Consistent with our genome analysis, we found that only *E. coli, K. pneumoniae, K. aerogenes*, and *P. fluorescens* generated agmatine (Figure 4d) and putrescine in the bacterial medium (Figure 4e).

**Figure 5 (a):**
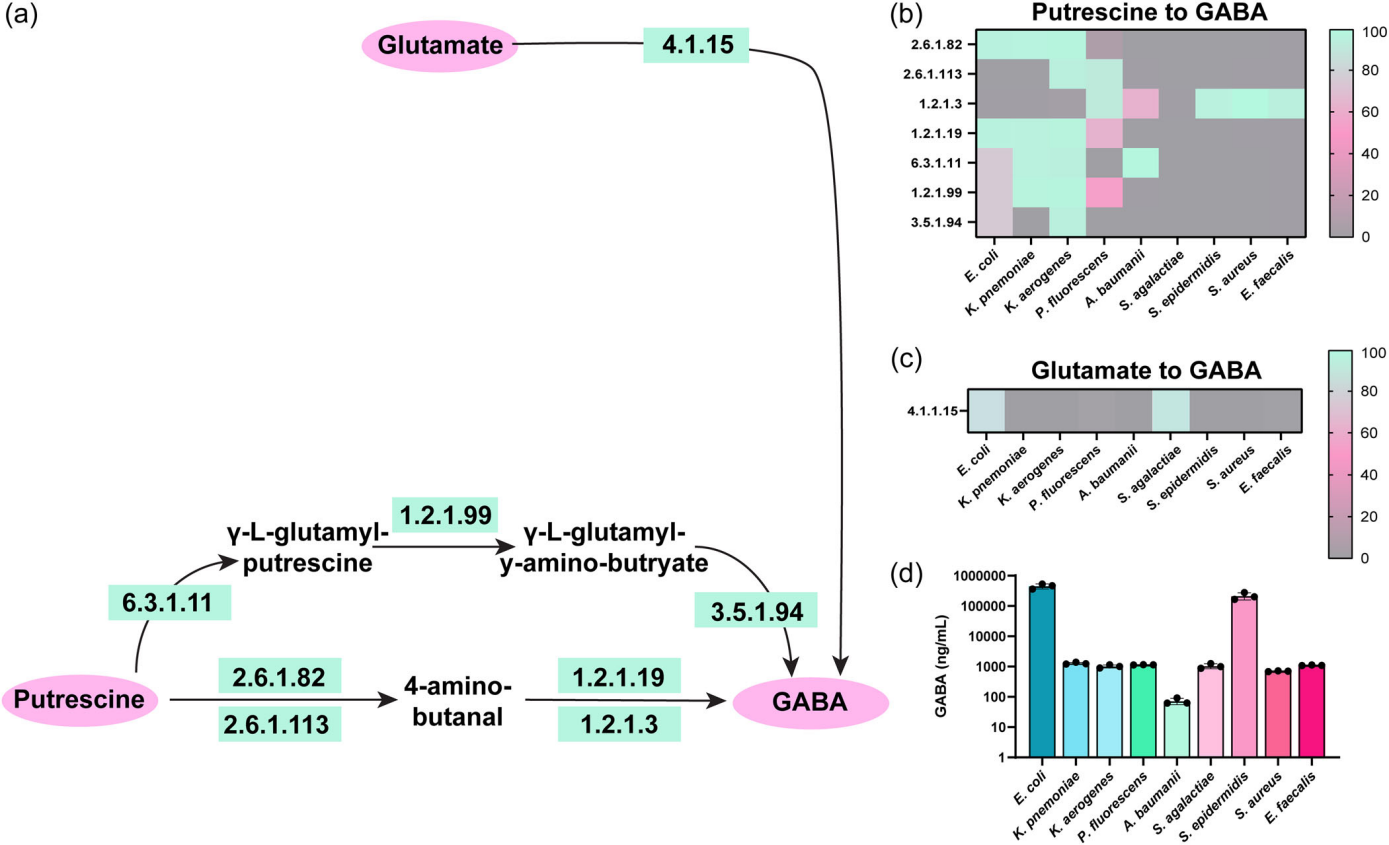
Simplified diagram of the pathways to convert Glutamate and Putrescine to GABA with the corresponding enzyme commission (EC) numbers responsible for these reactions. Percentage of bacterial genomes harboring ECs involved in the conversion of (b) Putrescine to GABA, and (c) Glutamate to GABA. Quantitative LC‐MS/MS‐based targeted metabolomics data of bacterial supernatant for the absolute concentrations of (d) GABA. *n* = 3; biological triplicate of bacteria samples. Each dot represents an individual data point.

Putrescine can be further converted to the neuro‐active compound GABA via a two‐step reaction (EC 2.6.1.82 (putrescine‐2‐oxoglutarate transaminase)/EC 2.6.1.113 (putrescine‐pyruvate transaminase) and EC 1.2.1.19 (aminobutyraldehydeehydrogenas)/EC 1.2.1.3 (aldehyde dehydrogenase)) or a three‐step reaction (EC 6.3.1.11 (glutamate‐putrescine ligase), EC 1.2.1.99 (4‐(γ‐glutamylamino)butanal dehydrogenase) and EC 3.5.1.94 (γ ‐glutamyl‐gamma‐aminobutyrate hydrolase)) (Figure 5a). We found that *E. coli, K. pneumoniae*, and *K. aerogenes* could convert putrescine to GABA through these pathways (Figure 5b). *A. baumannii, S. epidermidis, S. aureus*, and *E. faecalis* could generate GABA from 4‐amino‐butanal via EC 1.2.1.3. Glutamate can also be converted to GABA via EC 4.1.1.15 (glutamate decarboxylase), and we found that *E. coli* and *S. agalactiae* harbored this EC in their genomes (Figure 5c). Using LC‐MS/MS‐based targeted metabolomics we found that all our strains generated some GABA, with *E. coli* and *S. epidermidis* generating the highest concentrations (Figure 5d).

**Figure 6:**
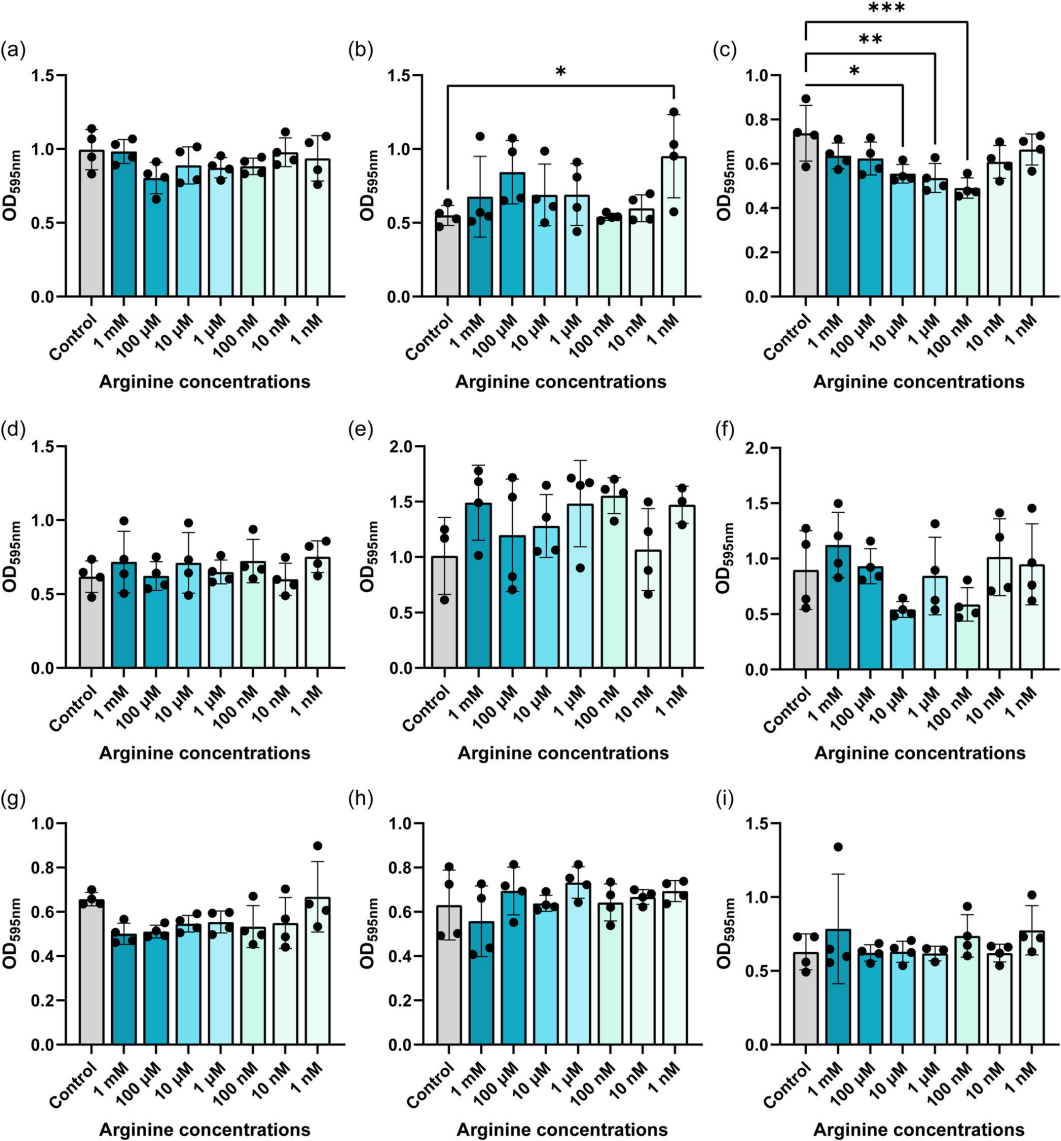
Bacteria were grown in the presence of a range of Arginine in tryptic soy broth (TSB) for 72 h and biofilm was assessed by crystal violet staining. The following bacteria were examined: (a) *Escherichia coli* ATCC BAA‐2452, (b) *Klebsiella pneumoniae* ATCC 9101, (c) *K. aerogenes* ATCC 13048, (d) *Pseudomonas fluorescens* CB1, (e) *Acinetobacter baumannii* ATCC 19606, (f) *Streptococcus agalactiae* ATCC 13813, (g) *Staphylococcus epidermidis* ATCC 51025, (h) *S. aureus* NCTC 12493, and (i) *Enterococcus faecalis* ATCC 29212. *n* = 4 wells for each bacterial biofilm analysis. Each dot represents an individual data point.

**Figure 7:**
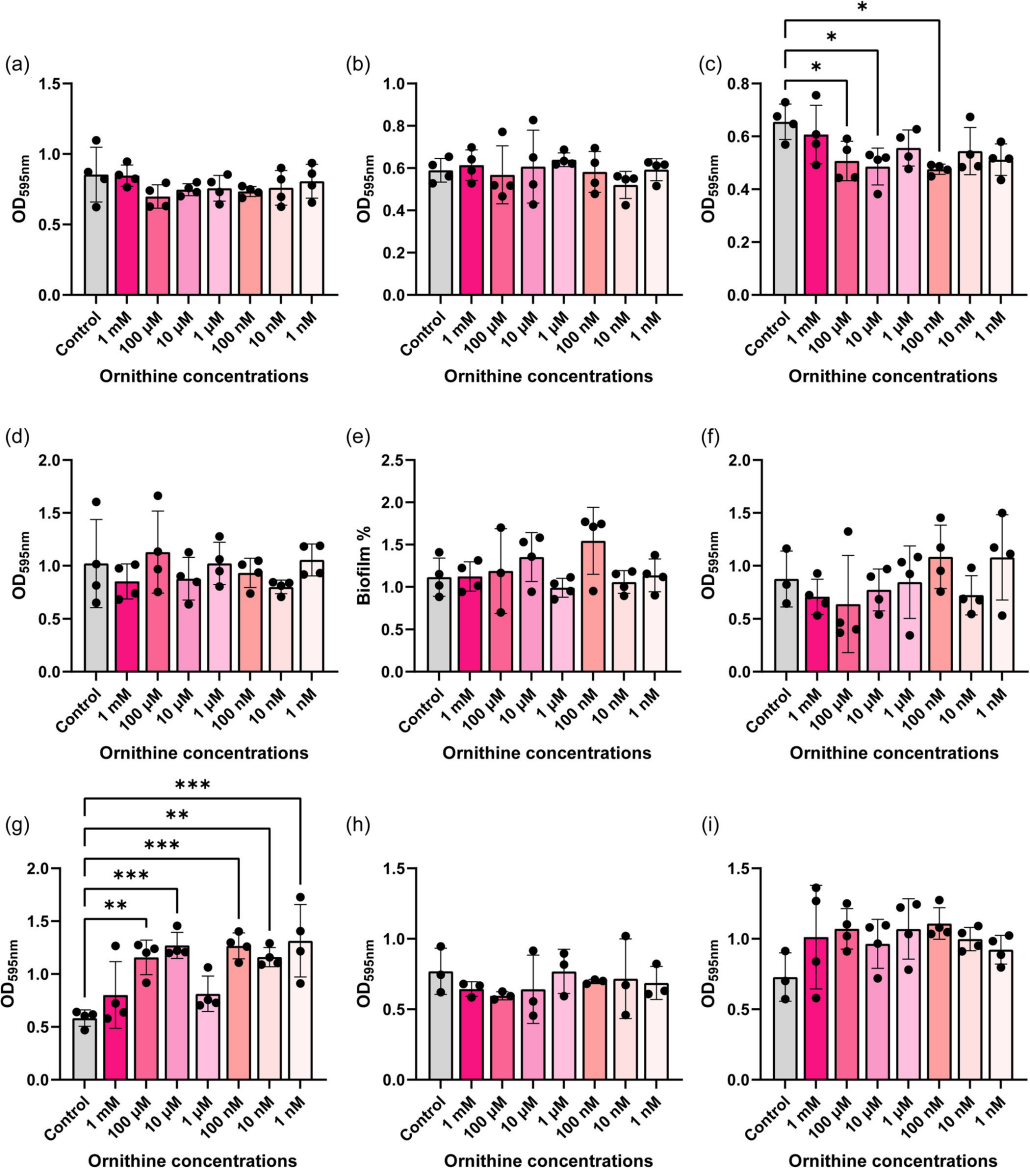
Bacteria were grown in the presence of a range of Ornithine in tryptic soy broth (TSB) for 72 h and biofilm was assessed by crystal violet staining. The following bacteria were examined: (a) *Escherichia coli* ATCC BAA‐2452, (b) *Klebsiella pneumoniae* ATCC 9101, (c) *K. aerogenes* ATCC 13048, (d) *Pseudomonas fluorescens* CB1, (e) *Acinetobacter baumannii* ATCC 19606, (f) *Streptococcus agalactiae* ATCC 13813, (g) *Staphylococcus epidermidis* ATCC 51025, (h) *S. aureus* NCTC 12493, and (i) *Enterococcus faecalis* ATCC 29212. *n* = 4 wells for biofilm analysis. Each dot represents an individual data point.

Arginine has been implicated in biofilm formation in several pathogens such as *Pseudomonas aeruginosa* (Drenkard, 2003; Scribani Rossi et al., 2022). To assess if arginine could modulate biofilm production in our gut pathobionts, we grew bacteria in Tryptic Soy Broth (TSB) with a range of arginine concentrations (1 mM–1 nM) and assessed biofilm formation by crystal violet staining (Figure 6). We found that 1 nM arginine elevated biofilm formation in *K. pneumoniae* (Figure 6b) and multiple concentrations of arginine suppressed biofilm production in *K. aerogenes* (Figure 6c). Interestingly, arginine did not impact the levels of biofilm production in our *E. coli, P. fluorescens, A. baumannii, S. agalactiae, S. epidermidis, S. aureus* and *E. faecalis* strains (Figure 6a, d–i). We also examined biofilm production in response to ornithine (Figure 7) and we observed decreased biofilm production in *K. aerogenes* in response to several concentrations of ornithine (Figure 7c). We also found increased biofilm production in response to almost all the concentrations of ornithine in *S. epidermidis* (Figure 7g), while the other bacteria did not exhibit changes in biofilm production in response to ornithine exposure. Collectively these data provide a more comprehensive perspective on arginine metabolism and biofilm responses in gut pathobionts.

## 7 DISCUSSION

L‐arginine is an amino acid that is converted by bacteria into a range of downstream metabolites, including citrulline, agmatine, ornithine, putrescine, and GABA. In this study, we examined arginine utilization and production of downstream compounds in diverse pathobionts found in the gastrointestinal tract. We found that both Gram‐negative (*E. coli, K. pneumoniae, K. aerogenes, P. fluorescens*, and *A. baumannii*) and Gram‐positive (*S. agalactiae, S. epidermidis, S. aureus, and E. faecalis*) pathobionts consumed arginine, glutamine, glutamate, and produced citrulline, ornithine and GABA under aerobic culture conditions; suggesting that these are conserved essential pathways within gut pathobionts. We found that *E. coli, K. pneumoniae, K. aerogenes*, and *P. fluorescens* also generated the arginine intermediate agmatine and *E. coli, K. pneumoniae, K. aerogenes*, and *P. fluorescens* produced the polyamine putrescine. Overall, these data suggest that arginine is an important amino acid for gut bacteria.

The arginine to citrulline to ornithine pathway, also known as the arginine deiminase (ADI) pathway, plays an important role in bacterial survival under acidic conditions in several pathogens (Degnan et al., 2000; Lindgren et al., 2014; Ryan et al., 2009; Xiong et al. 2014, 2016). The degradation of arginine generates ammonia, which can increase the acidic pH of the cellular cytoplasm compartment to a more neutral level and protect the cell from the potentially lethal effects of acidic extracellular environments (Allen & Bradley, 2011; Casiano‐Colón & Marquis, 1988; Degnan et al., 2000). Arginine is also an important regulator of virulence in *Salmonella enterica* serovar Typhimurium, EHEC, and *C. rodentium* (Choi et al., 2012; Menezes‐Garcia et al., 2020). In this study, we demonstrate that arginine degradation and citrulline and ornithine production is a conserved pathway in other pathobionts. We demonstrate that *E. coli, K. pneumoniae, K. aerogenes, P. fluorescens, A. baumannii*, *S. agalactiae, S. epidermidis, S. aureus, and E. faecalis* can all deplete arginine and secrete high concentrations of ornithine. Although all our selected bacteria generated ornithine, we found that the production of ornithine varied among our bacterial species. The highest concentrations of ornithine were generated by *K. pneumoniae*, *S. agalactiae, S. epidermidis, and E. faecalis*. We speculate that this observation could be due to increased ornithine transporters in these bacteria. Many bacteria possess L‐arginine/L‐ornithine exchangers, such as ArcD, ArcE, and ArgT, which take up L‐arginine and excrete L‐ornithine from the cytoplasm of the bacteria (Mallik et al., 2023; Noens & Lolkema, 2017). These transporters are present in Firmicutes and Proteobacteria; the phyla of our examined bacterial species. However, no paper to date has directly compared the levels and function of these ornithine transporters. Bacteria that have more active transport or simply have higher expression of these transporters could in theory secrete more ornithine. Another possibility is that the ornithine produced by *E. coli, K. aerogenes*, *P. fluorescens, A. baumannii*, and *S. aureus* may be used as a substrate for downstream metabolism. Ornithine can be used to generate putrescine or L‐glutamate 5‐seminaldehyde and low secreted levels of ornithine may reflect the shuttling of ornithine into other products. Finally, it is also possible that differences in ornithine may arise due to the involvement of enzymes that are outside the scope of our present analysis. For example, *S. agalactiae* contains roughly 8% hypothetical proteins in its genome which may play a role in ornithine production. In the future, we may perform stable‐label isotope tracing studies on this collection of pathobionts to ascertain all of the routes by which they can convert arginine to ornithine and more definitively identify additional downstream products of ornithine.

Our data indicates that *E. faecalis* ATCC 29212, *K. pneumoniae* ATCC 9101, and *S. epidermidis* ATCC 51025 consumed the most arginine and generated the highest amounts of ornithine. Previous work has demonstrated that *E. faecalis* generated ornithine promotes other pathobionts (Hunt et al., 2023; Keogh et al., 2016; Smith et al., 2022). L‐ornithine was found to stimulate enterobactin production and iron transfer in *E. faecalis* and uropathogenic *E. coli* polymicrobial biofilms (Keogh et al., 2016). Depletion of arginine and generation of ornithine by *E. faecalis* was found to enhance toxin production by the gut pathogen *Clostridioides difficile* and exacerbate intestinal inflammation in a mouse model (Smith et al., 2022). Consistent with these findings, another study found that arginine‐ornithine metabolism was a top pathway in individuals colonized by *C. difficile* (Pruss et al., 2022). *E. faecalis* also significantly increased uropathogenic *E. coli* biofilm growth and survival in vitro and in vivo in a mouse wound infection model by exporting L‐ornithine (Keogh et al., 2016). Ornithine was specifically found to facilitate *E. coli* biosynthesis of the enterobactin siderophore, allowing *E. coli* growth and biofilm formation in iron‐limiting conditions that would otherwise restrict its growth. *E. faecalis* generated ornithine was also found to promote arginine biosynthesis in another gut pathobiont *Proteus mirabilis* (Hunt et al., 2023). To date, no studies have examined the cross‐talk of ornithine from *K. pneumoniae* or *S. epidermidis* on other bacteria, but studies in the future would be valuable.

In this study, we identified that *E. coli, K. pneumoniae, K. aerogenes*, and *P. fluorescens* generated significant levels of putrescine, with *P. fluorescens* producing the highest concentration. Interestingly, we were unable to detect putrescine from our other bacterial supernatants. There are several interpretations of these findings. First, it is possible that A*. baumannii*, *S. agalactiae, S. epidermidis, S. aureus, and E. faecalis* do not generate putrescine. Our genome analysis suggests that the majority of annotated genomes from these bacterial groups do not possess the enzymatic machinery to produce putrescine. However, there may be other strains of these organisms that are not in the IMG database that do possess the enzymes to generate putrescine. For example, one study found that *S. aureus* strains BAA‐44, ATCC 43300, and ATCC 25293 were able to generate putrescine (Seravalli & Portugal, 2023). This study found that *S. aureus* ATCC 25293 was able to generate the highest concentration of putrescine; suggesting that some strains may be better at generating and secreting this product. Another possibility is that our growth conditions do not favor putrescine. We grew our selected pathobionts in a rich chemically defined medium in the laboratory setting. This environment may not put the appropriate stressors on the bacteria to force putrescine production. Another possibility is that all the putrescine generated by *A. baumannii*, *S. agalactiae, S. epidermidis, S. aureus, and E. faecalis* could be shuttled into the production of other compounds such as GABA. We detected GABA in the supernatant of all these bacterial strains so it's possible that these microbes were not actively secreting putrescine and instead were using it for the intracellular production of other downstream compounds. Finally, putrescine detection may have been affected by the accumulation of unmeasured metabolites, such as N‐acetylputrescine—a metabolite that has not yet been included in our assay. In the future, we plan to generate targeted approaches to measure multiple downstream targets of putrescine and more fully address this question.

Of interest, we identified that *E. coli* produced robust quantities of agmatine; far higher than any of the other bacteria strains. Agmatine has attracted attention in recent years as a candidate agent for the treatment of depression and neuropathic pain (Piletz et al., 2013; Suzuki et al., 2023). *E. coli* has been shown to efficiently generate agmatine and some groups have engineered *E. coli* to overexpress arginine decarboxylase on the extracellular surface of cells to create agmatine for commercial use. The L‐arginine/agmatine antiporter AdiC has been identified in *E. coli* and it plays a role in arginine‐dependent acid resistance (Ilgü et al., 2021). This acid resistance system in *E. coli* relies on the consumption of intracellular protons through the decarboxylation of L‐arginine to agmatine, which maintains a pH conducive to cell survival. The produced agmatine is then exchanged for external L‐arginine; thereby providing a new amino acid for the system (Ilgü et al., 2021). According to UniProt, AdiC is found in several Enterobacteriaceae, like *Klebsiella, Escherichia*, and *Pseudomonas*, but it is not found in *A. baumannii*, *S. agalactiae, S. epidermidis, S. aureus*, or *E. faecalis*. We speculate that high levels of agmatine in our Proteobacteria reflect the presence of AdiC and we hypothesize that *E. coli* may possess more AidC than our strains that secreted agmatine.

Another notable finding was that although all our pathobionts had the gene pathways to generate GABA, we observed varying levels of GABA production by our selected organisms. For example, we observed high levels of secreted GABA ( > 100,000 ng/ml) in supernatant from *E. coli* and *S. epidermidis*. In contrast, we observed only moderate levels of GABA ( ~ 1000 ng/ml) in cultures of *K. pneumoniae, K. aerogenes, P. fluorescens, S. agalactiae, S. aureus*, and *E. faecalis*. In other organisms, particularly lactic acid bacteria, the decarboxylation of glutamate results in the stoichiometric release of GABA and the consumption of a proton; leading to a more optimal pH in the bacterial cytosol (Dhakal et al., 2012). It is possible that *E. coli* and *S. epidermidis* generate more GABA to regulate their internal pH. Another study examining *Bacteroides* also reports large variability in GABA production among different species (Otaru et al., 2021). This study found that although multiple *Bacteroides* species (*B. caccae, B. dorei, B. faecis, B. intestinalis, B. ovatus, B. thetahiotaomicron, B. uniformis B. vulgatus*, and *B. xylanisolvens*) had glutamate decarboxylase (GAD)‐systems, the concentration of secreted GABA ranged from ~0.1 mM to 61 mM (Otaru et al., 2021). Using *B. thetaiotaomicron* as a model organism, they also noted that GABA secrtion started at the end of the exponential growth phase and rapidly increased over time, suggesting a nongrowth‐associated production (Otaru et al., 2021). It is possible that our pathobionts, particularly our *Klebsiella* species, may differ in their growth profiles and may not generate GABA at the same rate. Additionally, it is possible that GABA could be catabolized to succinate by the GABA shunt. Succinic semialdehyde dehydrogenases have been reported in bacteria like *Klebsiella pneumonia* (Sanchez et al., 1989) so GABA in these organisms may not be actively secreted and may instead be used to fuel other pathways.

One of the virulence factors that arginine and ornithine are known to modulate is biofilm production. Bacteria biofilms are widely recognized as a contributor to infectious diseases. Nassar et al. found that the genes *rocD* (ornithine aminotransferase) and *gudB* (glutamate dehydrogenase) are upregulated at both early and mature stages of biofilm formation in *S aureus* (Nassar et al., 2021); suggesting that arginine depletion and ornithine production are important regulators of biofilm. Our data indicated that *S. aureus* also generated high levels of ornithine, but it did not respond to external ornithine with biofilm production. However, we did observe significant biofilm production in the closely related species *S. epidermidis* in the presence of several concentrations of ornithine. Arginine has also been shown to enhance biofilm production in *Streptococcus mutans (*Vaziriamjad et al., 2022 *)*. We did not observe enhancement of biofilm in our pathobiont of interest *S. agalactiae* in response to supplemented arginine, but we did observe an increase in biofilm in *K. pneumonia*e at the lowest concentration of arginine. Interestingly, the related *K. aerogenes* showed a decrease in biofilm production in the presence of both arginine and ornithine. Previous reports indicate that arginine increases the killing of *K. pneumoniae* by neutrophils (Peñaloza et al., 2020). Thus, *K. aerogenes* supplemented with arginine or ornithine may affect its ability to be killed by the host by altering biofilm production and should be investigated in future studies. Additionally, it is possible that in most of our organisms, arginine and ornithine are used to generate other metabolites and not used for biofilm production.

This work highlights the utility of using bacterial genomes to build targeted metabolomics methods with authentic analytical (unlabeled) and stable‐label internal standards. In this study, we used this system to identify bacteria compounds in key pathways and perform quantitatively measure arginine, agmatine, citrulline, ornithine, N‐carbamoylputrescine, putrescine, glutamate, glutamine, and GABA in biologically relevant sample matrices. We have previously employed this workflow to understand other bacterial processes such as the production of neurotransmitters and short‐chain fatty acids (Fultz et al., 2021; Horvath et al., 2022, 2023a; Luck et al., 2021). We believe this approach could be used to tackle multiple bacterial pathways. However, there are several limitations to this study. Using the current system, we are unable to definitively identify which compounds are directly due to the consumption of arginine. To address this, we would need to employ stable‐label isotope tracing studies with ^13^C‐arginine‐supplemented cultures and identify the existence or incorporation of ^13^C‐atomic labels into downstream metabolic products. This data demonstrate that several gut pathobionts possess robust arginine pathways and we plan to perform these types of assays in the future to more definitely identify the arginine pathways in these organisms. Another limitation is that we are using mono‐cultures. This method is a good way to define what individual bacteria are capable of generating, but it fails to recapitulate the gut environment. In the future, we plan to examine bacterial co‐cultures and use stool‐based bioreactors to identify how bacterial cross‐feeding of arginine influences the levels of these compounds in an environment that better mimics the gut.

Our coupled approach of genome analysis and LC‐MS/MS‐based targeted metabolomics highlights the utility of combining these two techniques to examine bacterial metabolism. Our work indicates that arginine, citrulline, and ornithine metabolism are conserved traits among diverse pathobionts found in the gut. We speculate that these pathways may be regulating many of the features of these bacteria. As a result, these compounds may be targetable for limiting these pathobionts and promoting intestinal homeostasis. We believe that examining the secreted products of enteric bacteria is particularly important since these compounds can directly influence the host. Over the past two decades, multiple studies have identified that the metabolites of the gut microbiota play crucial roles in modulating host metabolism, production of neurotransmitters, barrier function, gut motility, regulation of inflammation, and the cross‐talk between the gut and other organs (Engevik et al., 2021a, 2021b; Gasaly et al., 2021; Gutierrez et al., 2023; Horvath et al., 2022; Horvath et al., 2023b; Liu et al., 2022; Zheng et al., 2022). We propose that examination of the secreted products of enteric pathobionts may shed light on their potential to disrupt normal intestinal functions. L‐arginine metabolism in particular has been shown to play a pivotal role in both bacterial and host metabolism (Nüse et al., 2023) and we predict that future studies examining bacterial arginine metabolism and host responses, using gnotobiotic animals or intestinal organoid platforms, will be highly informative.

## AUTHOR CONTRIBUTIONS

**Ian M Lillie**: Conceptualization (lead); Data curation (lead); Formal analysis (equal); Methodology (equal); Writing—original draft (lead); Writing—review & editing (equal). **Charles E Booth**: Formal analysis (equal); Investigation (equal); Visualization (equal); Writing—review & editing (equal). **Adelaide E Horvath**: Data curation (equal); Formal analysis (equal); Writing—review & editing (equal). **Matthew Mondragon**: Data curation (equal); Formal analysis (equal); Writing—review & editing (equal). **Melinda A Engevik**: Formal analysis (equal); Funding acquisition (equal); Supervision (equal); Visualization (equal); Writing—review & editing (equal). **Thomas D Horvath**: Conceptualization (equal); Formal analysis (equal); Methodology (equal); Supervision (equal); Writing—review & editing (lead).

## CONFLICT OF INTEREST STATEMENT

None declared.

## ETHICS STATEMENT

None required.

## Data Availability

The data that support the findings of this study are available in Zenodo: https://zenodo.org/doi/10.5281/zenodo.10794948.
